# Ionic liquid assisted extraction induced by emulsion breaking for extraction of trace metals in diesel, gasoline and kerosene prior to ICP-OES analysis

**DOI:** 10.1016/j.heliyon.2024.e26605

**Published:** 2024-02-17

**Authors:** Njabulo S. Mdluli, Philiswa N. Nomngongo, Nomvano Mketo

**Affiliations:** a Department of Chemistry, College of Science and Engineering and Technology, Florida Science Campus, University of South Africa, Roodepoort, 1710, Johannesburg, South Africa; b Department of Chemical Sciences, University of Johannesburg, PO Box 17011, Doornfontein 2028, Johannesburg, South Africa

## Abstract

This study describes a novel and greener ionic liquid assisted extraction induced by emulsion breaking (ILA-EIEB) method for extraction of As, Ba, Cd, Cr, Ni Pb, Sb, Sn, Tb, Te and V in fuel oils. The most influential extraction parameters were ionic liquid concentration [(1-Ethyl-3-methylimidazolium bis (trifluoromethylsulfonyl)], HNO_3_ concentration, Triton X-100 concentration, and sample mass and were optimised by using full factorial and Box-Behnken designs. The optimum conditions obtained were 0.035 % 1-Ethyl-3-methylimidazolium bis (trifluoromethylsulfonyl) concentration, 18 % v/v HNO_3_ concentration, 15 % w/v Triton X-100 concentration, and 0.1 g sample mass. The emulsions were fully broken by using a controlled heating water bath at temperature of 80 ± 2 °C for 30 ± 4 min, followed by centrifugation at 3500 rpm for 15 min. Under the optimum conditions, the proposed ILA-EIEB method was accurate (80.1–101 %) and precise (1.9–4.7 %) for all the investigated metals. The method detection limits were 0.107, 0.013, 3.494 and 0.560 μg/g for Ba, Na, Ni and V, respectively. The optimised ILA-EIEB method was then applied in real fuel samples and metal concentration levels ranged from 0.072 to 8.610 μg/g, which were consistent with other literature reported work. Therefore, this study suggests that the examined metal ions present in fuel oils commercialised in Johannesburg, South Africa are in tolerable concentration levels and are not a threat.

## Highlights

• A greener ionic liquid extraction induced by emulsion breaking was successfully developed.
• 1-Ethyl-3-methylimidazolium bis (trifluoromethylsulfonyl) assisted EIEB was reported for the first time.
• Acceptable accuracy (80–101%) and precision (1.9–4.7%) were obtained.
• Method detection limits (0.013–3.494 μg/g) were quite comparable with literature reports.
• The studies fuel oils contained metal ions at tolerable concentration levels (0.072–8.610 μg/g).

## 1 Introduction

Metals in fuel oils are known to cause corrosion of the processing metallic equipment, clogging of the engine filters, catalyst poisoning during catalytic cracking and severe atmospheric pollution [[1], [2], [3], [4], [5]]. It is reported that human exposure to these metals is associated with several health challenges such as liver and kidney damage (Zn, Pb, Co and Cu), carcinogenic cells (As, Cd and Ni), female infertility (Sb) and neurotoxic dysfunctionality (Co, Hg, Cr and Pb) [[6], [7], [8], [9]]. Metals like V and Ni degrade the quality of the oil, thereby affect the value negatively [10]. Due to the detrimental effects of the metals in fuel oils, there is a need to develop trustworthy and effective methods for the determination of concentration levels of metals in fuel oil for monitoring and awareness purposes.

Inductively coupled plasma (ICP) based instruments are well-documented for the determination of multielement in various matrices [11,12]. The most standard and easily available ICP techniques are inductively coupled plasma-optical emission spectroscopy (ICP-OES) and inductively coupled plasma-mass spectrometry (ICP-MS) [13,14]. It is worthy to indicate that entry level of ICP-OES is more cost-effective as compared to ICP-MS [15]. However, standard ICP-OES can only analyse aqueous samples with trace levels of organic and acid content [11]. Therefore, complexed matrices like fuels require sample preparation to convert them to aqueous phase [[16], [17], [18]]. Moreover, some of the metals in fuel oils are in trace levels (ppb-ppt) and require preconcentration step [12]. The most popular extraction and preconcentration methods reported for determination of metal ions in fuel oils are solid phase extraction (SPE) and liquid-liquid extraction (LLE) [[19], [20], [21], [22], [23], [24]]. The limitations with SPE is the prolonged separation step of the target analyte from the sorbent [17,25]. Moreover, some adsorbents are less selective towards the metals and require functionalization step, which increases analysis cost [26]. On the other hand, liquid-liquid micro-extraction (LLME), and extraction induced by emulsion breaking (EIEB) are the most reported LLE methods for extraction of metals in oily matrices [9,[27], [28], [29], [30]]. The use of EIEB has gained more popularity, because a total of 45 publications were reported from 2010 to 2022 [19,31]. The EIEB requires surfactant to improve the proper mixing of the two immiscible liquids (organic and aqueous) by reducing the surface tension [32]. A surfactant is an organic substance that has both the hydrophilic and hydrophobic functional groups [33]. The different surfactants reported in literature are Triton X-100 [19,[34], [35], [36]], Triton X-114 [19,21,36,37],Tween 20, Tween 40 and Tween 80 [32] with various properties. The Triton X-114 and Triton X-100 can give stable emulsions which results in very good recoveries [32,36]. However, Tween 20, Tween 40 and Tween 80 were reported in few publications, because they form very stable emulsion that take forever to break [32]. For metal extraction, HNO_3_ was mostly used as compared to HCl [17]. The use of acids helps metallic cations, so that they can be displaced from the basic sites of organic molecules by the H^+^ ions [17]. It is worthy to indicate that oils with high viscosity do not perform well for the extraction of metals using EIEB [38]. This is because, high viscosity oils get stuck on the container walls, thereby limit the formation of emulsion with the aqueous phase [39]. To overcome this challenge, some researchers have introduced carcinogenic organic solvents (toluene, hexane, and xylene) as diluents, however, too diluted oils would result in unstable emulsions, thereby reduce extraction recoveries [17,31].

Therefore, the aim of the current study is to develop ionic liquid assisted extraction induced by emulsion breaking (IA-EIEB) for extraction and preconcentration of As, Ba, Cr, Cd, Ni, Pb, Sb, Sn, Tb, Te and V in fuel oils, followed by inductively coupled plasma-optical emission spectroscopy (ICP-OES) analysis. The use of the ionic liquid is key in ensuring the stability of the formed emulsions, because the less stable the emulsions, the less contact time between the two phases and that lowers extraction efficiencies [40]. According to the authors knowledges, the use of ionic liquid to overcome the challenges of unstable emulsions when using EIEB is reported for the first time for extraction of metals in fuel oils..

## 2 Experimental procedures

### 2.1 Reagents and glassware

Plasticware that were used for the entire experiments were beakers, volumetric flasks and measuring cylinders. All the plasticware were soaked in soapy water for 3 h and then washed and rinsed with deionized water. After rinsing with deionized water, plasticware were soaked in 5% HNO_3_ solution for efficient removal of any metals stuck on the walls, prior to oven drying at 35 °C for 12 h. The reagents that were used included, 100 mg/L multielement standard for metals (As, Al, Ba, Be, Ca, Cd, Co, Cr, Fe, Ga, K, Li, Mg, Mn, Na, Ni, Pb, Sb,Se, Sn Sr,Tb, Te, V and Zn), 70 % ACS grade nitric acid, 5 mL of 1-Ethyl-3-methylimidazolium bis (trifluromethylsulfonyl) and standard reference material for fuel oils (NIST1634c) and were all purchased from Sigma-Aldrich, South Africa. The real fuel oil samples (gasoline, diesel, and kerosene) were purchased from different fuel filling stations around Johannesburg, South Africa. The nylon microfilters (0.45 μm) were purchased from Anatech Instrument, South Africa.

### 2.2 Instrumentation

A vortex mixer from Velp Scientifica in Italy was used for blending the solutions of surfactant, fuel oil, nitric acid, and xylene to form a homogenous mixture. A controlled water bath from Lab Tech (Sep Scie) manufactured in Korea was used to break the emulsions at 80 ± 2 °C. To ensure maximum phase separation, the tabletop centrifuge branded, NEYA16R from India was used. The extracted metals in an aqueous solution were analysed using Agilent Technologies 700 Series inductively coupled plasma-optical emission spectroscopy (ICP-OES). Sample uptake for the ICP-OES was accomplished by using an Agilent Technologies SPS 3 autosampler, and sample introduction was through concentric nebulizer and cyclonic spray chamber. Instrumental parameters were power (1.2 kW), which was maintained at the manufacturer's suggested levels, plasma (Ar) flow rate, nebulizer flow rate, peri-pump speed, and auxiliary gas flow rate, which were maintained at 1.5 L/min, 0.754 L/min, 15 rpm and 15.0 L/min, respectively. The analytical spectral wavelength lines best suitable for the investigated metals were As 188.980 nm, Ba 234.759 nm, Cd 214.439 nm, Cr 206.550 nm, Ni 216.55 nm, Pb 283.30 nm, Sb 217.582 nm, Sn 189.925 nm, Tb 350.914 nm, Te 190.802 nm and V 292.299 nm.

### 2.3 Ionic liquid assisted-extraction induced by emulsion breaking (ILA-EIEB) procedure

For efficient extraction of metals in fuel oil, a sample mass of 0.1 g was weighed and diluted with 500 μL of *p*-xylene in 15 mL centrifuge tubes. The diluted sample was then mixed with 5 mL of 18 % HNO_3_ and 0.035 % of 1-Ethyl-3-methylimidazolium bis(trifluoromethylsulfonyl) ionic liquid. Thereafter, 2 mL of Triton X-100 (15 %) was added, the mixture was thoroughly vortexed for 3 min and left undisturbed for 15 min to allow interaction of the immiscible phases. After 15 min, the emulsions were broken by heating the test tubes in a controlled water bath of 80 ± 2 °C for 30 ± 4 min. For ensuring maximum phase separation, a 15 min centrifugation step was carried out at a speed of 3500 rpm. Then, the aqueous phase was extracted using Eppendorf micropipette, transferred into a 10 mL volumetric flask and diluted to the mark. The extracts were then pass through microfilters and transferred into 15 mL centrifuge tubes for ICP-OES analysis.

### 2.4 Multivariate optimization of ILA-EIEB

Multivariate optimization has gained attention due to its capabilities of reducing the number of experiments needed to obtain the optimum conditions [41,42]. Therefore, the multivariate optimization approaches were used for the determination of the influential parameters affecting extraction of metals from fuels using ILA-EIEB. The investigated parameters were sample mass, ionic liquid [1-Ethyl-3-methylimidazolium bis(trifluoromethylsulfonyl)] concentration, Triton X-100 concentration and HNO_3_ concentration. The full factorial design (2^n^) was used for screening of significant parameters and Box- Behnken design for further optimization of significant parameters. The maximum and minimum values for the full factorial design were 10–20 %, 5–20 %, 0.02–0.05 % and 0.05–0.2 g for nitric acid, Triton X-100, 1-Ethyl-3-methylimidazolium bis(trifluoromethylsulfonyl) and sample mass, respectively. Minitab 2018 statistical software was used for generating experiments and interpreting data obtained from both experimental designs (two-level full factorial and Box- Behnken).

## 3 Results and discussion

### 3.1 Multivariate optimization of the ILA-EIEB

#### 3.1.1 Full factorial design

**Fig. 1:**
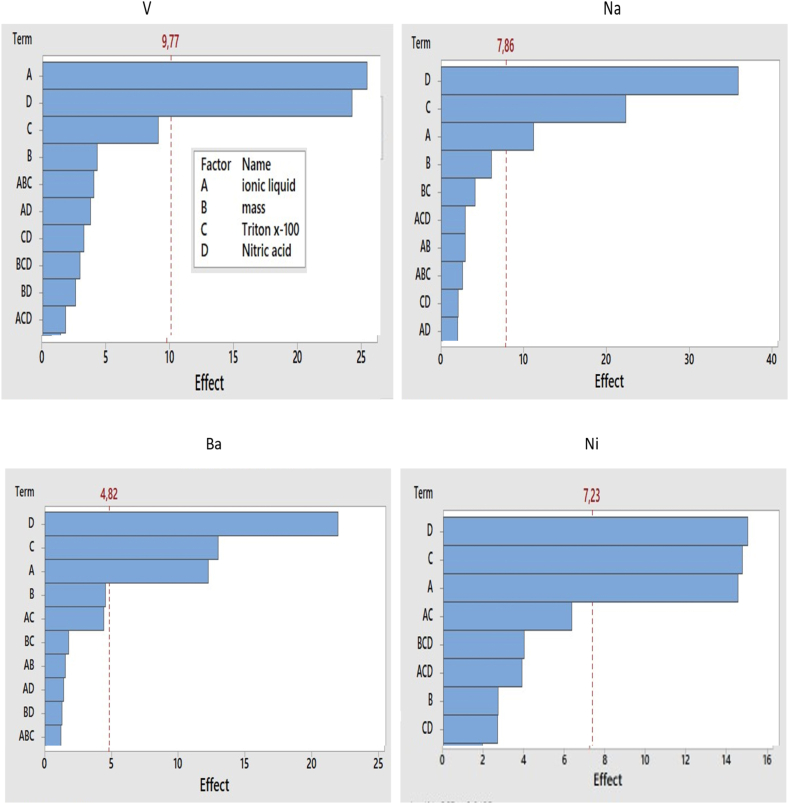
(A–D): Pareto chart for a level 2- full factorial design (2^4^) at 95 % confidence level for optimization of sample mass, nitric acid, ionic liquid, and Triton x-100 concentration for extraction of Ba, Na, Ni and V using ILA-EIEB (n = 3).

Table S1 shows that there were four parameters (concentrations of HNO_3_, Triton X-100, ionic liquid, and sample mass) that were investigated using full factorial design (FFD). Pareto charts were generated to assess parameters which were significant at 95 % confidence level (see Fig. 1A–D). Results from Pareto charts shows that at 95 % confidence level, HNO_3_ concentration, Triton X-100 concentration, and ionic liquid concentration were the most significant parameters. Moreover, Table S1 illustrate acceptable percentage recoveries of up to 100 ± 6 % for Ba, Na, and Ni and 85.4 % for V. The low recoveries for V are due to the inability of the proposed ILA-EIEB to extract +5-oxidation state (VO_3_^−^) of V from the organic matrix. [10]. The three significant parameters (concentrations of HNO_3_, Triton X-100, and ionic liquid) were further optimised by using BBD and 0.1 g sample mass was selected to be optimum.

#### 3.1.2 Box- Behnken design (BBD)

**Fig. 2:**
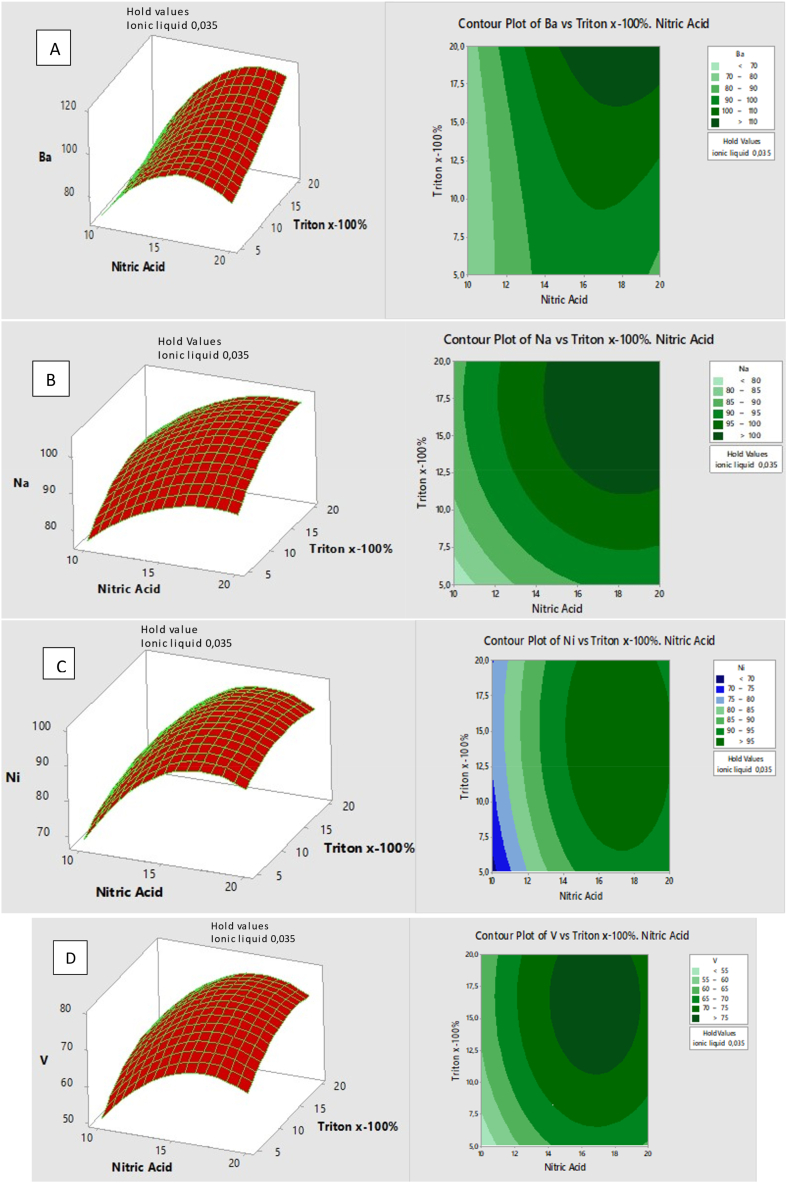
A–D: Response surfaces for Ba, Na, V and Ni Versus Time. Temperature obtained from Box-Behnken design. Experimental conditions: 0.1g of the sample and all the other factors were varied (n = 3).

The Box- Behnken was opted for further optimization as literature indicated that it is among the most convenient response surface methodologies (RSMs) for parameters that are from three and upwards [30]. This design was constructed so that a total of 15 experiments were produced from a single block design (Table S2). The most ideal experimental conditions of ILA-EIEB were also supported with quadratic equations (Equation S1 A-D) produced by response surface methodology. The surface plots and the contour plots are presented in Fig. 2 A-D. The plots show that increasing Triton X-100 concentration from 5 to 15 % and HNO_3_ concentration from 10 to 15 % showed a significant increase in percentage recoveries for Na (see Fig. 2A). Therefore, the optimal conditions for ILA-EIEB were 0.035 % 1-Ethyl-3-methylimidazolium bis(trifluoromethylsulfonyl), 18 % HNO_3_, 15 % Triton X-100, and 0.1 g sample mass, as shown by the plots and quadratic equations. The outcomes of the BBD were validated using the analysis of variance (ANOVA). From the ANOVA, the *p*-values for Ba, Ni and V were 0.565, 0.113 and 0.546, respectively, confirming that the data for the three metals was a perfect fit to the RSM model. However, the *p*-value for Na was 0.011, implying a nonfit of data. Therefore, Na was considered as an outlier. Moreover, when considering the adjusted R^2^, the values for Ba, Na, Ni and V were ranging from 0.8698 to 0.9315 which indicated that the model can satisfactorily explain data variability.

#### 3.1.3 The role of ionic liquid in the newly developed ILA-EIEB

**Fig. 3:**
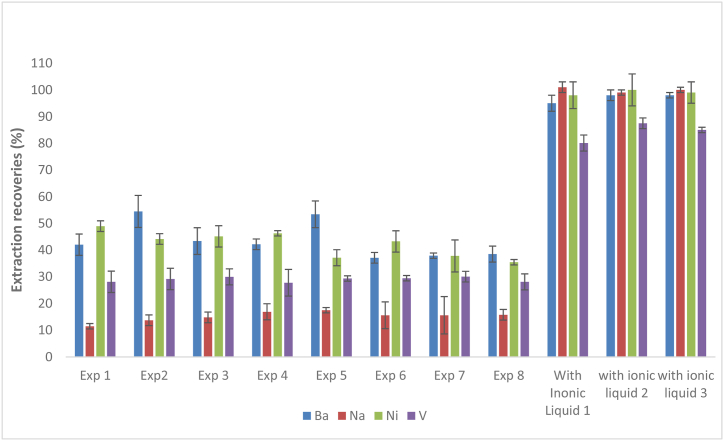
Experiments to compare the importance of ionic liquid in extraction of metals from fuel oils.

The limitation that arises when using organic solvent was the formation of emulsions that were not stable [20].]. Therefore, the addition of 1-Ethyl-3-methylimidazolium bis(trifluoromethylsulfonyl) ionic liquid helped to create much stable emulsions. The stable emulsions are a key in ensuring that much contact time between the organic and aqueous phase is achieved [32]. A total of 11 experiments were then conducted to investigate if there were any effect of the presence of the ionic liquid in the extraction effeciences. Out of the 11 experiments, 8 were conducted without the addition of an ionic liquid while 3 were conducted with the addition of ionic liquid. It was observed that all the emulsions formed without ionic liquid were broken from 1 to 3 min and the average percentage recoveries were less than 20 % for Na 35 % for V, 40 % for Ba and 60 % for Ni (see Fig. 3). However, when the ionic liquid was used, much improved recoveries (80–101 %) were obtained as illustrated in Fig. 3.

#### 3.1.4 Method validation using NIST1634c

**Fig. 4:**
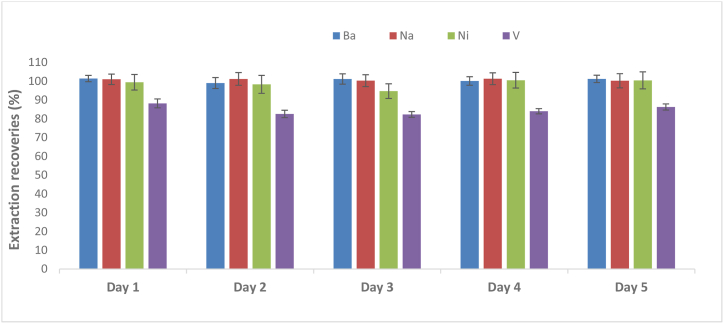
Method Validation using a standard method for EIEB and NIST1643c. Extraction conditions 0.2 g (NIST1634c), 1 mL xylene, 5% Triton X-100 and 10% HNO_3_ (n = 5).

The method reported by de Castro et al. 2016 was followed for the validation of ILA-EIEB using the NIST1634c [38]. A total of 5 runs were conducted over a period of 5 days (interday precision data) and Ba, Ni and Ni reported acceptable extraction recoveries and precision ranging from 94.7 to 101.4% and 1.3–4.8%, respectively. However, V showed a slight decrease in the recoveries (82.3–88.2%) as illustrated in Fig. 4. The recoveries for V were then compared with other literature reports. For example, a study reported by de Sousa and co-workers investigated the use of EIEB for the determination of Ni and V by GF-AAS in off-shore Brazilian crude oils and very low recoveries (50-74%) of V ranging were obtained [10]. Additionally, a *t*-test confirmed that V cannot be extracted quantitatively from the crude oil samples using the EIEB procedure. Therefore, the challenges of V extraction in fuel oils when using EIEB should be considered in future studies.

### 3.2 Analytical figures of merit

The analytical figures of merit were investigated by using the optimum conditions generated by the RSM. The method detection limit (MDL), method quantification limit (MQL), correlation coefficient (R^2^), and sensitivity (gradient) were the analytical figures that were considered for examination. The R^2^ displayed excellent linearity because it ranged from 0.9983 to 0.9997 for all the investigated metals. Moreover, Na recorded lowest MDL of 0.013 μg/g, suggesting that the developed ILA-EIEB method was mostly nsitive for Na. However, Ni, on the other hand, demonstrated relatively high MDL of 3.494 μg/g, meaning that it was the least sensitive metal. The lower sensitivity might be due to the fact that Ni securely bound to the organic molecules in the oil via a strong covalent bond [23]. Table 1 shows preconcentration factors (PFs) for Ba, Na, Ni, and V, which were calculated to be 87.5, 100, 50, and 30, respectively.. The obtained PFs were improved as compared to literature reports (32).

**Table 1:** Analytical features of the EIEB method for quantitative extraction of Ba, Na, Ni and V in NIST1634c: EIEB conditions; ionic liquid (0.035%), Triton X-100 (15%) and HNO_3_ (18%) replicates (n = 3).

Metal	SDV of intensity (cps)	Sensitivity (cps L μg^−1^)	LOD (μg/L)	LOQ (μg/L)	MLOD (μg/g)	MLOQ (μg/g)	Accuracy (%)	Precision (%)	Correlation coefficient R^2^
Ba	2.71	3.0299	2.6833	8.9441896	0.107	0.357	95	3.2	0.9983
Na	5.59	515.407	0.0325374	0.108458	0.013	0.043	101	4.7	0.9991
Ni	3.14	1.0783	8.736	29.11	3.494	13.12	98	1.9	0.9990
V	1.67	3.5801	1.3994023	4.6647	0.560	1.866	80.1	3.3	0.9997

### 3.3 Comparison of the proposed ILA-EIEB with literature reports

Table 2 was designed to compare the newly developed ILA-EIEB method with other EIEB methods reported fromliterature. It is important to note that the limit of detection (LOD) was chosen as one of the figures used to assess the merits of the ILA-EIEB, since it is an analytical parameter that is closely related to sensitivity. The proposed ILA-EIEB method showed 2.68, 0.03, 8.74 and 1.399 μg/L LODs for Ba, Na, Ni and V, respctively, which were quite comparable with literature reported studies [[10], [32], [37], [43], [44], [45]]. Furthermore, the newly developed ILA-EIEB method was accurate (80-101) and precise (1.9-4.7) for all the invesigated metals (Ba, Na, Ni and V). It is worthy to indicate that, several studies have reported on EIEB, where high volumes of carcinogenic organic solvents (xylene and hexane) and concentrated corrosive acids (HNO_3_ and HCl) were used, making the extraction environmentally unfriendly [35,[43], [46], [47]]. It was also noted that, the extraction of V was challenging, and these shortcomings were reported in literature, where extraction effeciencies of 50–74% were obtained [10]. However, the proposed ILA-EIEB showed an improved extraction efficiency (80%) of V in in fuel oils. Therefore, the ILA-EIEB can be considered as the best alternative for extraction of metals in crude oil fractions such as gasoline, diesel and kerosene samples, since the obtained preconcentration factors (87.5, 100, 50 and 30) were five times better than the literature report [32].

**Table 2:** Comparison of analytical figures of merit between ILA-EIEB and other EIEB reported from literature.

Fuel matrix	Metal	Detection Technique	Reagents	LOD (μg/L)	Preconcentration Factor (PE)	Accuracy (%)	Precision (%)	Extraction time and emulsion breaker	Sample preparation	Ref
Bitumen	Ni	GFAAS	1.5 mL Toluene, 40% w/v Triton X-100 and 40% v/vHNO_3_	9	NA	100	<10	Water bath 60 °C for 120 min	EIEB	[44]
Crude oil	Na	ICP-OES	0.8g Triton X-100, 0.6 mL xylene and 1 mL conc. HNO_3_	9.8	NA	92.8–102.2	3.5–10.9	Water bath for 5 min at 80 °C	EIEB	[43]
Diesel	Ni	EAAS	7% w/v Triton X-100, 10 mL diesel and 10% v/v HNO_3_	145	5	85.2–109	8.8	Water bath 80 °C	EIEB	[32]
Off-shore Brazilian crude oil	Ni and V	GF-AAS	20% (w/v) Triton X-114 and 6.5 mol L^−1^ HNO_3_	NA	NA	Ni (82–103%) and V (50–74%)	NA	Water bath at 90 °C for 40 min and centrifugation for 15 min for 500 rpm	EIEB	[10]
Diesel	Ni	ICP-MS	20% w/v Triton X-114, 2 mL conc. HNO_3_	0.07	NA	84–113	<3.30	Water bath 90 °C	EIEB	[37]
Lubricating oil	Ni and V	ETAAS	0.02 mL Triton X-100, 0.05 mL conc. HNO_3_ and 0.11 mL	0.77 and 0.83 μg/g	NA	94–115	<7	Water bath 88 ± 2 °C	EIEB	[45]
NIST1634c	Ba, Na, Ni and V	ICP-OES	15% w/v Triton X-100 18%v/vHNO_3,_ 0.035% ionic liquid	2.68, 0.03, 8.74 and 1.399	87.5, 100, 50 and 30	95, 101, 98 and 80.1	3.2, 4.7,1.9 and 3.3	Water bath 80 ± 2 °C for 30 ± 4 min	ILA-EIEB	This work

**NB**: Conc. is referring to Concentrated.

### 3.4 Application of extraction ILA-EIEB in real fuel samples

The ILA-EIEB sample preparation method was applied in diesel, gasoline, and kerosene samples for determination of metals by ICP-OES. Table 3 shows that the amount of As was very low in all the investigated fuel oil samples. The measured values were 0.084–0.116 μg/g for gasoline, 0.084–0.116 μg/g for kerosene, and 0.1–0.25 μg/g for diesel. The As concentration levels were also compared to other studies and it was discovered that the As in South African is not that high [20,34,48]. Cassella and co-workers reported the EIEB for extraction of Al, Cu, Mn, Ni, Sn, and V in diesel oil followed by ICP-MS [23]. In comparison to the current study, the reported concentrations of Sn were quite low ranging from 1.55 to 1.86 μg/L. With regards to barium, there was almost no difference in the concentration in the different fuel samples and the levels were rangingbetween 2.95 and 8.7 μg/g. Moreover, Te concentration levels ranged from 0.067 to 0.590 μg/g in all samples, the levels were significantly low as compared to the other metals investigated in the current study. It is important to note that many of the examined elements, such as Sb, Te, Ge, and Ba, were not previously reported in literature using EIEB for extraction of metals in fuel samples.

**Table 3:** Concentration levels of metal ions expressed as μg/g in the samples of, diesel, gasoline, and kerosene (A, B and C) after extraction using ILA-EIEB and analysis by ICP-OES.

Element	Diesel samples (μg/g)			Kerosene samples (μg/g)			Gasoline samples (μg/g)		
	A	B	C	A	B	C	A	B	C
As	0.25	0.21 ± 0.0	0.100 ± 0	0.1 ± 0.00	0.08	0.95 ± 0.0	0.104	0.12 ± 0.0	0.084 ± 0.
Ba	7.3 ± 0.0	7.4 ± 0.2	8.0 ± 0.2	8.9 ± 0.5	6.8	6.9 ± 0.5	8.0	2.95 ± 0.1	8.7 ± 0.4
Cr	5.9 ± 0.1	2.1 ± 0.06	1.9 ± 0.04	2.3 ± 0.1	1.8 ± 0.05	1.3 ± 0.1	1.1 ± 0.08	0.9 ± 0.02	1.2 ± 0.01
Cd	4.1 ± 0.08	3.0 ± 0.07	5.1 ± 0.2	5.0 ± 0.07	0.8 ± 0.02	0.58 ± 0.08	5.01 ± 0.1	4.1 ± 0.2	3.1 ± 0.4
Ni	1.0 ± 0.01	1.0 ± 0.01	1.25 ± 0.1	1.54 ± 0.0	1.83 ± 0.1	1.8 ± 0.1	2.1 ± 0.1	2.09 ± 0.06	2.3 ± 0.05
Pb	6.35 ± 0	6.9 ± 0.1	5.89 ± 0.5	8.61 ± 0.3	5.81	4.2 ± 0.1	5.87	7.1 ± 0.6	5.4 ± 0.2
Sb	1.03 ± 0.	0.548	0.37 ± 0.0	0.94 ± 0.01	0.92	0.89 ± 0.0	0.53	1.0 ± 0.1	1.0 ± 0.01
Sn	1.14 ± 0	1.14 ± 0.0	0.925±	0.645 ± 0.0	0.64	0.64 ± 0.0	0.810	0.513 ± 0.	1.5 ± 0.08
Tb	0.541 ± 0.	0.487 ± 0	0.483±	0.075 ± 0.0	0.067	0.072 ± 0.	0.466	0.590 ± 0.	0.53 ± 0.0
Te	2.12 ± 0	3.8 ± 0.01	3.95 ± 0.1	0.95 ± 0.0	0.92	0.85 ± 0.0	<DL	1.54 ± 0.1	2.12 ± 0.1
V	1.21 ± 0.01	1.81 ± 0.1	1.65 ± 0.1	1.43 ± 0.2	1.1 ± 0.1	1.3 ± 0.1	1.09 ± 0.06	1.8 ± 0.1	2.3 ± 0.2

## 4 Conclusion

A greener ionic liquid assisted- extraction induced by emulsion breaking (ILA-EIEB) was successfully developed for the extraction of As, Ba, Cd, Cr, Ni Pb, Sb, Sn, Tb, Te and V in diesel, kerosene and gasoline samples. The method reported accepted MDLs (0.013–3.494 μg/g), accuracy (80.1–101 %), preconcentration factors (30–100) and precision (1.9–4.7 %). The full factorial (FFD) and Box-Behnken designs (BBD) were used to investigate the most optimum conditions for the effecient extraction of metals in fuels using ILA-EIEB method. Both FFD and BBD showed optimum conditions of 0.035%, 15% w/v, 0.1g and 18% w/v for ionic liquid concetration, Triton X-100 concentration, sample mass and HNO_3_ concentration, respectively. The ionic liquid provided a solution to the traditional EIEB's unstable emulsion issues. When the concentrations of theinvestigated metals were compared to reports in the literature, it was clear that the locally purchased fuel oils are not a threat to the vehicle engine.

## Data availability

All data generated is included in article and/or in supplementary materials.

## CRediT authorship contribution statement

**Njabulo S. Mdluli:** Writing – original draft, Validation, Methodology, Investigation, Formal analysis. **Philiswa N. Nomngongo:** Writing – review & editing, Visualization, Supervision, Conceptualization. **Nomvano Mketo:** Writing – review & editing, Visualization, Supervision, Resources, Project administration, Funding acquisition, Conceptualization.

## Declaration of competing interest

The authors declare the following financial interests/personal relationships which may be considered as potential competing interests: Nomvano Mketo reports financial support was provided by South African National Research Foundation.
