# Supplementary material for: Ionic liquid assisted extraction induced by emulsion breaking for extraction of trace metals in diesel, gasoline and kerosene prior to ICP-OES analysis
Source: Heliyon. 2024 Feb 17;10(5):e26605. doi: 10.1016/j.heliyon.2024.e26605 (PMC10912242; doi:10.1016/j.heliyon.2024.e26605)
Supplement: Multimedia component 1 [file mmc1.docx]

**Ionic liquid assisted extraction induced by emulsion breaking as a preconcentration procedure prior to ICP-OES analysis for the determination of trace metals in crude oil, diesel, kerosene and gasoline samples**

Njabulo S. Mdluli^a^, Philiswa N. Nomngongo^b^, Nomvano Mketo^a^^[[1]](#footnote-1)^

*^a^Department of Chemistry, College of Science and Engineering and Technology, Florida Science Campus, University of South Africa, Roodepoort, 1710, Johannesburg, South Africa*

*^b^Department of Chemical Sciences, University of Johannesburg, PO Box 17011, Doornfontein 2028, Johannesburg, South Africa.*

**Table S1:** The effect of varying sample mass, ionic liquid , nitric acid and Triton X-100 concentration during ILA-EIEB on NIST1364c to achieve high percentage recoveries of Ba, Na, Ni and V. Replicates (n=3)

| Exp | Ionic liquid (%) | Mass (g) | Triton x-100 (%) | Nitric acid (%) | Ba (%R) | Na (%R) | Ni (%R) | V (%R) | Ba  (%RSD) | Na  (%RSD) | Ni  (%RSD) | Ni (%RSD) |  | |
| --- | --- | --- | --- | --- | --- | --- | --- | --- | --- | --- | --- | --- | --- | --- |
| 1 | 0.02 | 0.05 | 5 | 10 | 67.8 | 40.9 | 76.6 | 51.0 | 14.5 | 17.1 | 9.2 | 18.1 | |  |
| 2 | 0.05 | 0.05 | 5 | 10 | 50.8 | 30.2 | 55.7 | 17.60 | 16.3 | 19.2 | 14.2 | 15.2 | |  |
| 3 | 0.02 | 0.10 | 5 | 10 | 75.8 | 43.6 | 80.1 | 63.40 | 10.5 | 17.5 | 8.14 | 14.3 | |  |
| 4 | 0.05 | 0.10 | 5 | 10 | 56.5 | 32.4 | 64.0 | 31.7 | 16.2 | 10.2 | 16.7 | 12.9 | |  |
| 5 | 0.02 | 0.05 | 20 | 10 | 76.8 | 68.4 | 87.7 | 67.90 | 8.3 | 7.1 | 6.2 | 5.4 | |  |
| 6 | 0.05 | 0.05 | 20 | 10 | 67 | 43.9 | 78.5 | 37.7 | 7.3 | 8.4 | 7.7 | 3.4 | |  |
| 7 | 0.02 | 0.10 | 20 | 10 | 81.7 | 69.0 | 87.9 | 72.2 | 5.4 | 4.3 | 5.2 | 5.9 | |  |
| 8 | 0.05 | 0.1 | 20 | 10 | 72.5 | 62.3 | 69.8 | 43.8 | 6.8 | 6.1 | 8.2 | 2.3 | |  |
| 9 | 0.02 | 0.05 | 5 | 20 | 86.1 | 80.1 | 91.3 | 80.60 | 3.4 | 4.6 | 4.8 | 5.1 | |  |
| 10 | 0.05 | 0.05 | 5 | 20 | 75.4 | 67.0 | 69.5 | 62.30 | 5.1 | 5.2 | 5.0 | 6.7 | |  |
| 11 | 0.02 | 0.10 | 5 | 20 | 96.3 | 81.3 | 93.8 | 83.50 | 2.0 | 1.9 | 1.3 | 2.5 | |  |
| 12 | 0.05 | 0.10 | 5 | 20 | 76.8 | 69.4 | 70.4 | 54.40 | 4.8 | 4.9 | 4.4 | 5.1 | |  |
| 13 | 0.02 | 0.05 | 20 | 20 | 100.2 | 93.1 | 96.0 | 84.80 | 1.2 | 2.4 | 3.5 | 3.8 | |  |
| 14 | 0.05 | 0.05 | 20 | 20 | 94.2 | 85.2 | 93.5 | 61 | 6.4 | 6.5 | 6.9 | 7.1 | |  |
| 15 | 0.02 | 0.10 | 20 | 20 | 101.4 | 101.8 | 103.3 | 85.4 | 2.3 | 2.7 | 1.9 | 4.7 | |  |
| 16 | 0.05 | 0.10 | 20 | 20 | 94.1 | 98.2 | 100.2 | 72.2 | 3.5 |  |  |  | |  |

**Table S2:** Response surface optimization for Ionic-liquid, Triton X- 100 and HNO_3_ for high recoveries of Ba, Na, Ni and V. Replicates (n=3)

| Experiment | Ionic-  liquid | Triton x-100 | HNO_3_ % | % R  Ba Na Ni V | | | | %RSD  Ba Na Ni V | | | |
| --- | --- | --- | --- | --- | --- | --- | --- | --- | --- | --- | --- |
| Exp 1 | 0.02 | 5 | 10 | 83.3 | 84.4 | 88.9 | 65.6 | 6.2 | 4.7 | 5.3 | 7.1 |
| Exp 2 | 0.05 | 5 | 10 | 87.7 | 85.4 | 89.3 | 68.8 | 5.1 | 4.6 | 5.4 | 6.9 |
| Exp 3 | 0.02 | 20 | 10 | 95.4 | 94.1 | 94.6 | 69.1 | 4.7 | 3.1 | 5.1 | 6.5 |
| Exp 4 | 0.05 | 20 | 10 | 103.7 | 100 | 95.1 | 72.0 | 4.9 | 2.1 | 4.3 | 5.8 |
| Exp 5 | 0.02 | 5 | 20 | 45.4 | 79.8 | 62.7 | 56.6 | 6.7 | 6.3 | 3.1 | 4.9 |
| Exp 6 | 0.05 | 5 | 20 | 62.9 | 81.6 | 77.2 | 56.9 | 6.3 | 4.8 | 5.6 | 8.1 |
| Exp 7 | 0.02 | 20 | 20 | 90.9 | 99.9 | 97.9 | 69.6 | 4.2 | 2.9 | 1.8 | 4.7 |
| Exp 8 | 0.05 | 20 | 20 | 98.9 | 100 | 98.5 | 75.3 | 1.9 | 2.5 | 1.8 | 3.1 |
| Exp 9 | 0.01 | 12.5 | 15 | 74.8 | 79.4 | 72.9 | 49.2 | 5.1 | 4.8 | 3.9 | 6.1 |
| Exp 10 | 0.06 | 12.5 | 15 | 82.2 | 90.5 | 78.2 | 64.9 | 4.7 | 4.6 | 4.1 | 5.0 |
| Exp 11 | 0.035 | 12.5 | 15 | 80.4 | 88.0 | 86.5 | 60 | 3.9 | 4.3 | 3.8 | 4.8 |
| Exp 12 | 0.035 | 12.5 | 17.5 | 111.3 | 100 | 88.6 | 74.6 | 1.9 | 0.8 | 1.4 | 4.9 |
| Exp 13 | 0.035 | 12.5 | 17.5 | 103.8 | 98.7 | 97.3 | 76.3 | 2.2 | 1.3 | 4.1 | 5.1 |
| Exp 14 | 0.035 | 15 | 17.5 | 99.1 | 99.2 | 96.8 | 75 | 1.3 | 2.6 | 4.9 | 4.8 |
| Exp 15 | 0.035 | 12.5 | 17.5 | 99.8 | 98.7 | 96.0 | 74.4 | 3.2 | 0.6 | 3.6 | 4.2 |

Supplementary equations from Eq.1A-1D

$$Ba=-145.4+3965A-2.47B+21.07C-46667A^{2}+0.0373B^{2}-0.633C^{2}+8.4AB-32.3AC+0.157BC \boldsymbol{Eq.1.A}$$

$$Na=-6.9+1064A+2.03B-7.63C-15148A^{2}-0.0748B^{2}-0.2043C^{2}+13.3AB-6.0AC+0.0120BC \boldsymbol{Eq.1.B}$$

$$Ni=-90.4+1173A+2.25B+17.75C-4907A^{2}-0.0650B^{2}-0.461C^{2}+0.20AB-46.7AC-0.0213BC \boldsymbol{Eq.1.C}$$

$$V=-49.4+475+2.69+11.23C-8907A^{2}-0.0774B^{2}-0.3482C^{2}-0.7AB+17.0AC-0.0073BC \boldsymbol{Eq.1.D}$$

1. Corresponding author: Email address: nomvano.mketo@gmail.com or mketon@unisa.ac.za; Tel: +27114712032 [↑](#footnote-ref-1)
